# Clients’ subjective experiences of navigating challenges in Gestalt therapy: an interpretative phenomenological analysis

**DOI:** 10.3389/fpsyt.2025.1593918

**Published:** 2025-10-27

**Authors:** Raphaela Elisabeth Kaisler, Yvonne Schaffler

**Affiliations:** ^1^ Department of Psychology, Bertha von Suttner Private University St. Pölten, St. Pölten, Austria; ^2^ Fachsektion Integrative Gestalttherapie, Österreichischer Arbeitskreis für Gruppentherapie und Gruppendynamik (ÖAGG), Vienna, Austria; ^3^ Department for Psychosomatic Medicine and Psychotherapy, University for Continuing Education Krems, Krems, Austria

**Keywords:** Gestalt therapy, personality structure, OPD, interpretative phenomenological analysis, qualitative research

## Abstract

**Introduction:**

This study explored the lived experiences of four female clients with common mental disorders and suboptimal structural integration after 30 sessions of Gestalt therapy, navigating emotional and relational challenges, bodily awareness, and agency.

**Method:**

Using Interpretative Phenomenological Analysis (IPA), we analyzed four semi-structured interviews, followed by a cross-case comparison.

**Results:**

Two experiential patterns emerged. Clients with low-integrated personality structures faced significant difficulties with emotional regulation, self-concept stability, and relational dynamics. They relied heavily on external validation and struggled to develop cohesive internal processes. While they described increased awareness of emotions and bodily sensations, these experiences remained fragmented and did not translate into broader self-integration. In contrast, clients with moderate-integrated personality structures demonstrated greater emotional regulation and a more stable self-concept. However, despite engaging in reflective processing and linking past experiences to present challenges, they continued to struggle with self-agency.

**Discussion:**

Findings highlight the distinct ways in which clients with varying levels of personality integration experience Gestalt therapy and suggest that while emotional and relational growth occurred, deeper structural transformation remained limited. This study underscores the need for tailoring therapeutic interventions to clients’ structural integration levels, particularly by addressing the challenges of fostering agency in structurally vulnerable clients.

## 1 Introduction

Gestalt therapy is a holistic, process-oriented approach grounded in humanistic psychotherapy that has been explored for its potential effectiveness in treating various psychological disorders (1–8) while also fostering emotional growth, self-awareness, and relational improvements (9). It views individuals as integrated entities whose physical, mental, and cognitive components are intricately linked to their social and environmental contexts (10, 11). By focusing on the present moment and experiential awareness, Gestalt therapy aims to enhance an individual’s capacity to navigate emotional and relational challenges (2, 4, 12, 13).

Gestalt therapy emphasizes *structural integration* as a foundation for psychological health, encompassing the ability to regulate emotions, sustain a coherent sense of self, and engage in stable relational dynamics (14). Clients with lower personality integration often struggle with emotional regulation, self-organization, and maintaining stable relational dynamics. The Operationalized Psychodynamic Diagnostics offers a structured approach to assessing personality structures by capturing past and current relational experiences (15). It provides deeper insight into an individual’s self-development throughout treatment (16) and assesses structural functioning, defined as “the availability of psychic functions necessary for the organization of the self and its relationships with internal and external objects” (15, p.54). Understanding and addressing structural functioning in therapy can help foster an empowered and integrated self (17).

From a structural perspective, personality integration is closely linked to narrative identity−the internalized and evolving life story that helps individuals make sense of past experiences, present challenges, and future aspirations (18). When narrative identity is fragmented, as in clients with lower personality integration (19, 20), emotions and relational experiences remain disconnected from a broader self-concept, making it difficult to develop a stable sense of self. Gestalt therapy’s emphasis on making meaning of one’s experience and the surrounding world (21) by enhancing body awareness and imagination (2, 22), expressive capacities and therefore a sense of agency and fulfillment of intentionality for contact (23), as well as exploring dreams, dialogues and metaphors (12, 13), may facilitate the integration of fragmented self-experiences into a more coherent self-narrative.

Beyond fostering narrative integration, relational dynamics are pivotal in self-development and therapeutic change. Personal identity does not form in isolation but emerges through interactions with others, shaping and reinforcing an individual’s self-perception. Drawing from Martin Buber’s philosophy, the “I-Thou” relationship emphasizes authentic, mutual encounters that foster personal growth and self-understanding (24). Since Gestalt therapy is deeply rooted in relational experiences, embodied interactions and intersubjectivity further contribute to the development of self and identity (25–27). Gestalt therapy provides a framework for integrating self-experiences across different relational contexts (28) by engaging clients in direct relational encounters and emphasizing embodied awareness.

Although prior studies have examined the role of Gestalt therapy in fostering emotional growth and relational awareness (4, 9, 29), few have specifically investigated how clients with suboptimal personality integration experience and navigate challenges throughout their therapeutic journey. A previous multiple case study and companion paper to this study (30) found that while both groups reported improvements in emotional awareness, relational engagement, and subjective empowerment, their therapeutic processes and reliance on different coping strategies varied. Clients with low integration primarily engaged with therapy through bodily awareness and verbalization, whereas those with moderate integration focused more on relationship-oriented interventions and self-regulation. However, despite clients’ perceived improvements, no measurable change in personality functioning was observed after 30 sessions (30). This raises questions about how clients with suboptimal personality integration experience and interpret their therapeutic change, particularly concerning typical challenges with bodily awareness, narrative identity, relationships, and emotional regulation. While prior research has given hints on general outcome differences between structural integration levels (31), little is known about how these clients navigate the Gestalt therapeutic process, construct meaning from their experiences, and perceive progress.

This study explored the research question: How do clients with suboptimal personality integration experience, interpret, and navigate challenges since they sought Gestalt therapy? By analyzing these lived experiences, we aimed to gain deeper insight into how different levels of personality integration shape the processes of change within Gestalt therapy.

## 2 Method

### 2.1 Design

Building on the companion paper (30), this study explores the experiences of female clients with common mental disorders and suboptimal personality integration following 30 sessions of Gestalt therapy. The original study employed a mixed-methods design to examine therapy outcomes and processes in clients with varying levels of personality integration, finding improvements in mental health symptoms across all participants. Four German-speaking female clients participated in 60-minute semi-structured interviews, reflecting on their change processes after completing therapy. A content analysis of therapy diaries from clients and psychotherapists identified factors contributing to successful, empowering therapy outcomes. The coding system developed from these diaries was applied to the interview data and expanded with inductive categories. However, this analysis focused on the quantification of categories and did not capture the detailed experiential aspects of clients’ perceived change processes.

To address this gap, we conducted an in-depth re-analysis of the semi-structured interviews using Interpretative Phenomenological Analysis (IPA; 32, 33). The interviews explored clients’ experiences navigating challenges since beginning therapy and their ability to cope with them independently. Clients were thus asked about the bodily and emotional experiences associated with their sense of managing challenges on their own. They also reflected on pivotal moments in decision-making and their perceived impact, identifying the resources and competencies that supported them throughout their therapeutic journey. These aspects addressed also relational aspects – the relationship between the client and the therapist – by describing difficult situations during the therapy process. The interview guide included the following questions: 1) Please select and describe a difficult situation from the last few weeks (i.e., since starting therapy). How did you manage to overcome this situation? 2) Please share a situation from the last few weeks where you felt you could make a difference. What bodily sensations did you experience in that moment? If you feel this now, which emotions are involved? 3) Please discuss a situation in the last few weeks where you had to make an important decision. These interview questions explored how clients experienced and made meaning of dealing with challenges throughout Gestalt therapy beyond symptom improvement.

The IPA method is rooted in phenomenology, the philosophical study of experience, which serves as a central theoretical foundation for humanistic therapies (34), including Integrative Gestalt Therapy (IG; 35–37). IPA seeks to understand how individuals make sense of their lived experiences, uncovering the personal meaning and significance of these within their specific contexts. It is an experiential method grounded in three key philosophical traditions: phenomenology, hermeneutics, and idiography (32, p. 6). Given its focus on complex, deeply personal phenomena, IPA is particularly suited for examining psychological change processes in therapy.

### 2.2 Participants

IPA guidelines emphasize the need for a sufficiently homogeneous sample to facilitate the exploration of contextually specific yet theoretically meaningful insights. While homogeneity does not require identical participants, it entails a shared experience or perspective relevant to the research question (33). Studies typically include between four and ten interviews, and in this case, data from four participants were deemed sufficient, as their narratives were rich, reflective, and closely tied to the constructs under investigation. Given the study’s focus on in-depth, idiographic exploration, fewer cases allowed for a more detailed examination of individual meaning-making and change processes, aligning with IPA’s emphasis on depth over breadth (33).

The sample consisted of four Caucasian female clients who shared key demographic similarities, the experience of seeking psychotherapy for depressive symptoms, and perceived mental health improvements after 30 sessions of therapy. The participants met the criteria for common mental health disorder (depression, anxiety) and the personality structure was assessed by an OPD-2 diagnostic interview (2014) conducted by the therapist and certified OPD rater (see 30). Two participants had moderately integrated personality structures (*M_age_* = 30.00, *SD* = 1.41), while the other two had low-integrated personality structures (*M_age_* = 44.00, *SD* = 5.66); among the latter two, one participant had a comorbid borderline personality disorder diagnosis. All participants had higher education. For additional sociodemographic details, see Kaisler et al. (30).

### 2.3 Data analysis

We conducted an Interpretative Phenomenological Analysis (IPA; 32) on four interviews, focusing on the first 20 minutes of the transcribed semi-structured interviews. In this initial section, clients shared their experiences with challenging situations and their strategies to overcome them. They also discussed the difficulties they faced when making decisions, both during and beyond treatment. This selection was based on our research question regarding how clients experience growth and change throughout therapy. This was possible due to their improvements in mental health symptoms and suffering during Gestalt therapy. First, the first author reviewed all transcripts and marked notable nonverbal elements (pauses, laughter, changes in tone) to capture embodied aspects of meaning-making. The first author then analyzed each case individually, engaging in an idiographic, interpretative process. Initial exploratory notes included descriptive, linguistic, and conceptual observations. In the next step, experiential statements were formulated to encapsulate clients’ subjective experiences and psychological dynamics. These statements were then clustered to synthesize the progression of clients’ experiences throughout their narratives.

The final step in the individual case analysis involved creating a Personal Experiential Themes (PETs) table, where each cluster was labeled with a PET theme. Once all PETs were developed, the first and the second author iteratively discussed and refined clusters and subthemes to ensure interpretative plausibility and depth. **Supplementary Table 1** provides an overview of PETs for each case.

In the cross-case analysis, both authors collaboratively examined thematic convergence and divergence across the four cases, grouping clients’ PETs into Group Experiential Themes (GETs). At this stage, personality structures were not initially considered. However, during the interpretative process, all subthemes ultimately aligned with either moderate- or low-integrated personality structures, suggesting an emergent link between personality structure and change experience. **Supplementary Table 2** provides an overview of GETs and subthemes.

To enhance rigor, interpretations were discussed, refined, and checked against raw data to ensure faithfulness to participants’ lived experiences while acknowledging the researcher’s role in meaning-making. Quotes were translated into English for illustration.

## 3 Results

To explore the participants’ experiences of suffering and change throughout treatment, we analyzed Group Experiential Themes (GET) across the four cases using Interpretative Phenomenological Analysis (32). Detailed examples for clients’ themes for each GET are provided in **Supplementary Table 2** that are summarized in **Table 1** presenting the four main themes and corresponding subthemes. The theme “Idealization and Generalization” was specific to the two clients with low-integrated personality structures, while the remaining themes reflected overarching experiences shared by all clients. However, their manifestations within these themes differed, as captured in the subthemes.

**Table 1:** Group Experiential Themes (GET) and subthemes of Gestalt therapy process with clients with low- and moderate-integrated personality.

Themes	Personality structure
Idealization and generalization	low-integrated
Self-perception	
Devaluing oneself and others (black and white thinking)	low-integrated
Self-doubt and insecurities	moderately-integrated
Relieving yourself through others	
Navigating decision-making by external input	low-integrated
Resonance from others is helpful to make own decisions	moderately-integrated
Therapy progress	
Experiencing in the here and now	low-integrated
Relating and reflecting on the past and present	moderately-integrated

### 3.1 Idealization and generalization

This theme captures how clients construct their identities and perceive others through an idealized lens, which influences their ability to process complex situations. Clients often reported feeling overwhelmed in challenging situations by comparing their reality with an unattainable ideal, which generates distress and inner pressure.

For instance, Client 1, a low-integrated client, exhibits an idealized self-concept by filtering her perception of others through a rigid and unrealistic image (“I deny that others are as volatile as I am”), while simultaneously devaluing her own behavior (“we don’t, we are late”). She articulates this inner conflict when discussing her family dynamics:

“In my mind, everyone else who complains about their children doesn’t have the problems I have with my children, and I deny that others are as volatile in their work, thinking, and lifestyle as I am. I do know other people, but they don’t seem as mentally unstable to me as I am. [ … ] Well, I often say that the others are too perfect, and I don’t think that’s true at all [ … ] they [other families] are always on time and definitely have something with them [school supplies], and we don’t, and we are late and so”. (client 1)

Similarly, Client 4, also low-integrated, recounts a threatening encounter with a former partner turned stalker. She overgeneralizes her experience (*“how quickly you get into it”*) and reinterprets threatening situations (*“I’m not so badly affected myself”*), comparing her experience to that of other women in violent situations. This minimizes the severity of her own situation, reinforcing a sense of helplessness:

“So I counsel women who are affected by violence, and I’ve seen that I always have to say to them ‘I’m not so badly affected myself’ but in a different form [stalking]. And now you’re in there so quickly. [ … ] That’s how quickly you get into it. Suddenly you don’t know what to do anymore, exactly”. (client 4)

Rooted in the clients’ self-concept, the idealization of others and reinterpretation of situations limit their ability to respond adaptively. These patterns, which in our study only apply to low-integrated clients, create a kind of “emotional gridlock,” where distorted perceptions and defense mechanisms prevent them from taking effective action. As a result, they struggle to navigate challenging situations with clarity, reinforcing a cycle of avoidance and self-justification that hinders adaptation. These patterns were observed in clients with lower integrated structures representing one PETs with four subthemes for Client 1 and two PETs with seven subthemes for Client 4. However, we also observed these patterns for Client 2, which had moderate integration and consisted of one PET with four subthemes.

### 3.2 Self-perception

This theme explores how clients perceive themselves and their relationships, focusing on patterns of *(self-)*devaluation, self-doubt, and insecurity. While clients with low-integrated personality structures experience predominantly negative self-perception and low self-worth, clients with moderate integration struggle with self-doubt and uncertainty, particularly in interpersonal interactions.

#### 3.2.1 Devaluing oneself and others (black-and-white thinking)

This subtheme highlights how clients with low-integrated personality structures exhibit self-devaluation, often perceiving themselves as inadequate or incapable. Their narratives reveal a tendency to anticipate failure, avoid asserting themselves, and struggle with maintaining a stable self-concept.

Client 1 describes an ongoing struggle with self-doubt and identity instability:

“And I have the feeling that I am in a state of permanent puberty. And I often don’t know what I want today will be different tomorrow or I’ll talk myself into something or talk myself out of something, so it’s getting more and more bizarre what I think. And then I really must go away and let them do it because otherwise it escalates [family situation].” (client 1)

Her description of herself as being in a “state of permanent puberty” suggests an internalized sense of instability and self-doubt. She perceives herself as erratic and unreliable, reinforcing her negative self-concept. Her avoidant response (“I must go away and let them do it”) further illustrates how her self-perception translates into social withdrawal and passivity.

Similarly, Client 4 demonstrates a tendency to devalue her own knowledge and opinions, believing others to be more competent:

“I know my way around and I’m interested, but some things I just don’t get in politics. I’ve realized that I just don’t feel comfortable with people who say ‘you can’t do that [vote]’ [ … ] that I used to think that he and she are much more knowledgeable and read better articles, so that’s why I have to attach myself to them [opinion]”. (client 4)

Her statement reveals self-devaluation through comparison. By assuming that others are “much more knowledgeable,” she undermines her own abilities and relies on external validation, contributing to self-doubt and insecurity in decision-making.

Even in moments of achievement, clients struggled to internalize their successes, often minimizing their accomplishments or attributing them to external factors. Client 1 illustrates this pattern when reflecting on a work-related success:

“So it was like, let’s say, 30 people were contacted [project] to see if they wanted to take part and then I don’t know, four or five people got in touch to continue working on the topic [ … ] no one winded me up or anything else because I just got in front of them and I actually did very well. And what’s also nice is that I started by thinking I was afraid of the appointment because maybe all the unpleasant people would get in touch or maybe ahm I don’t know uh write back then go back please I’m not interested in that. But I was able to get a few people motivated and I think maybe they wouldn’t have done that with everyone”. (client 1)

Although the clients described successful situations where they achieved something meaningful and felt momentary emotional relief, they tended to devalue these achievements:

“A nice situation from a short time ago was a family reunion [ … ] but I mean, these are the kind of things where I thought ah yes, I mean it was a coincidence [borrowing toiletries to niece] that it was in the bag or the bag that I just had with me. But then I thought to myself actually well done and it was definitely needed in the situation. As a woman, you have to be prepared”. (client 1)

Clients both reported a negative self-image and low self-worth, which profoundly influenced how they perceived themselves and interacted with others. Client 1’s idealized self-conception made it difficult to accept discrepancies between her ideal self and reality. Client 4, similarly, struggled with low self-worth, which led her to devalue others to justify her own decisions. Together, these patterns of negative self-perception contributed to difficulties in social interactions, decision-making, and emotional well-being and concerned the two clients with low-integrated personality structures. In total, these patters were observed in three PETs with 11 subthemes of client 1 and two PETs with 11 subthemes of client 4.

#### 3.2.2 Self-doubts and insecurities

In contrast to low-integrated clients, those with moderate integration primarily struggled with interpersonal self-doubt and relational insecurities.

Client 2, for instance, describes hesitation in seeking support, fearing that doing so would burden others:

“I don’t want to impose myself [problems] on others, and that I make myself vulnerable with my weakness or with my issues, which are somehow present there, and I wasn’t quite sure how that works [getting in touch with others]”. (client 2)

Her words reveal a deep-seated fear of burdening others, reinforcing her reluctance to initiate or maintain social connections. The phrase “I wasn’t quite sure how that works” suggests not only confusion about building relationships but also uncertainty about whether she is entitled to emotional support.

Client 2 also describes a dilemma between authenticity and social belonging, struggling with how much of her true self to reveal:

“I really thought, well, I have to pretend so that people stay with me so that I don’t feel lonely and on the other hand I can’t show myself because otherwise they run away and that’s why I’m lonely and that I have been lonely for a very long time or that I still find it difficult to be honest with myself and to maintain honest relationships”. (client 2)

This statement illustrates a core relational conflict—the belief that authenticity will drive people away while pretending ensures social inclusion at the cost of emotional isolation. Her admission of long-term loneliness highlights how this insecurity shapes her relational patterns, preventing deeper, more fulfilling connections.

Client 3 similarly experiences self-doubt and hesitation in asserting personal opinions, particularly in social settings:

“I think that would actually be too much for the bride too [party], but somehow I don’t dare say that it’s too much, because it might come across as weird or something, ahm, and then somehow I didn’t really know what to do about it “. (client 3)

Her reluctance to express discomfort reveals a fear of social misjudgment, which leads to passivity and difficulty advocating for her own needs. The phrase “it might come across as weird” suggests a strong concern with how others perceive her, reinforcing self-doubt and emotional restraint.

Clients’ experiences in social situations reveal deep-seated insecurities rooted in fears of rejection, emotional exposure, and uncertainty about self-worth. Their struggles with authentic self-expression and concerns about how others perceive them lead to social hesitation and self-censorship. These patterns limit their ability to form meaningful connections, as the fear of negative evaluation often overrides their need for closeness. They remain caught in a cycle of relational uncertainty and emotional guardedness without a sense of social security. Overall, these patterns were observed in clients with moderate integration consisting of three PETs with 17 subthemes in Client 2 and one PET with six subthemes in Client 3.

### 3.3 Relieving yourself through others

This theme explores how clients navigate decision-making through a balance of external input and self-confidence.

#### 3.3.1 Navigating decision-making by external input

This subtheme highlights how clients rely on external influences to regulate decision-making anxiety. Rather than making choices independently, they seek validation and guidance from external sources, reducing the perceived emotional burden of decision-making. While both groups sought external perspectives, the two clients with low-integrated personality structures relied on external feedback for emotional regulation, whereas those with moderate integration used external input as a source of resonance rather than dependence.

Client 1 describes how external emotional cues influenced her choice:

“There was a chat message from a group [ … ] that was a 20-year reunion, and the chat message was full of positive vibes and then I thought to myself, don’t let it be ruined somehow and just go [conflict family situation]”. (client 1)

Here, her decision was shaped by the emotional tone of the group, illustrating a strong reliance on external validation rather than internal confidence. Similarly, she describes relief when decisions are made collectively:

“When you visit a city [with your family], you don’t always have to decide for yourself what to do. So that was also a big weight off my shoulders, that everyone should like it now”. (client 1)

Her phrase “big weight off my shoulders” reflects decision-making anxiety, suggesting that external input relieves the emotional stress of personal responsibility.

Similarly, Client 4 relied on the options of others in deciding for the upcoming election that helped her to make choices without external validation:

“I allowed myself to be distracted or made dependent on the opinions of people who I really appreciate and who know their stuff very well”. (client 4)

However, the external input and knowledge about the situation promoted their self-confidence that led to own decision-making, as client 1 described:

“I asked the children if they had any big objections to this holiday destination, and after nothing came [ … ] I suggested the destination [ … ] we would do it spontaneously, again because I said we would book something now and stay in Austria”. (client 1)

As clients’ self-confidence and knowledge increased, they became more capable of independent decision-making. This shift reflects a move from external to internal regulation, where decision security is no longer dependent on external validation but supported by self-trust and cognitive autonomy. Reflective evaluation enabled them to assess their choices critically, fostering a greater sense of agency and self-efficacy, ultimately leading to more autonomous and empowered decision-making. Only clients with low-integrated structure shared these themes, consisting of three PETs with twelve subthemes for Client 1 and two PETs with twelve subthemes for Client 4.

#### 3.3.2 Resonance from others is helpful to make own decisions

This subtheme explores how external input helps clients manage insecurities and gain confidence in making their own decisions. By seeking resonance from others, clients found relief and encouragement, which facilitated their ability to act independently.

Client 3 described her decision-making process when starting psychotherapy, illustrating how external cues and situational factors played a role in overcoming hesitation:

“I wanted to start therapy for a long time and then I just couldn’t bring myself to do it. And then there was another dicey situation with my parents, and then I thought, okay, actually I like my life the way it is anyway, and I don’t want to end my life just because it’s always difficult, and then I went shopping and looked to the right, and then I saw that there was a therapy practice, and then I thought, okay, well, that’s fate now “. (client 3)

Her decision to enter therapy was shaped by a mix of external circumstances and self-reflection, suggesting that the presence of external prompts helped her take action. The idea of “fate” reflects a psychological shift where external resonance provided a sense of direction, making the decision feel more attainable.

Additionally, receiving support in decision-making helped alleviate the pressure she felt, enabling her to act with greater confidence:

“My boyfriend doesn’t want to have children at the moment and we’ve actually been together for a little over four years now and we’ve always said that we want to have children and then somehow, out of the blue, he said he couldn’t actually imagine having a child because of the climate crisis and all the other crises in the world. That was a slap in the face for me and then we somehow decided to discuss it in therapy and I decided not to decide yet on what to do next “. (client 3)

Here, her partner’s shift in perspective created significant emotional uncertainty, but rather than making an immediate decision, she found comfort in deferring the choice and seeking external input through therapy. This highlights how resonance from others does not necessarily provide direct answers but offers a structured space for processing emotions and uncertainties.

Similarly, Client 2 developed a strategy to manage social anxiety and insecurity by using voice messages as a form of controlled communication. This approach allowed her to engage socially while minimizing immediate pressure, reinforcing her sense of security through positive feedback from others:

“I think I’m even more uninhibited because I don’t have this direct feedback [from the other person]. I can just chat freely, and if I get a voice message in return, I can choose when I listen to it and when I reply to it, and then I can organize my own resources a bit better”. (client 2)

By controlling the timing and structure of her interactions, she was able to reduce social anxiety and enhance her sense of agency in communication. The flexibility of asynchronous interaction provided a buffer against potential social stressors, helping her to build confidence in engaging with others.

Both clients actively sought external validation and support to navigate complex decisions and emotions. The resonance and feedback they received from others enabled them to move forward with greater confidence, underscoring the importance of external support in their decision-making processes. This reliance on external input was not merely about seeking approval but rather about creating a sense of psychological security, facilitating their ability to act with greater autonomy. In total, these themes were observed only in clients with moderately integrated personality structures, as shown in two PETs with nine subthemes for Client 2 and four PETs with 24 subthemes for Client 3.

### 3.4 Therapy progress

This theme explores the growth clients experience throughout the psychotherapy process, highlighting how their ability to process emotions and past experiences evolves over time. Clients with a lower level of integration reported greater awareness of emotions and bodily sensations in the present moment, suggesting an emerging ability to recognize, differentiate, and contextualize their feelings. In contrast, those with higher levels of integration reflected on their past experiences, identifying how these events influenced their present situations and emotional responses.

#### 3.4.1 Experiencing in the here and now

This subtheme focuses on how clients develop awareness of their emotions and bodily sensations in the present moment. By learning to observe and contextualize these experiences, they gradually gain a deeper understanding of their emotional states and how these influence their actions.

Client 1, with a low-integrated personality, described the pressure of making independent decisions, which initially felt overwhelming. However, in successful situations, she was able to differentiate between emotions and bodily sensations, demonstrating a heightened awareness of her internal experiences at work:

“There’s a certain sadness that I can’t go in more confidently [at work]. Then there’s a certain feeling of happiness that I managed to send the email, there’s a bit of satisfaction that when I said I’d take care of it, no one said, ah yes ‘you’ hm so it was the same ‘yes do it’. [ … ] that’s a kind of satisfaction that there’s already a trust in me now. [ … ] and of course I was afraid that someone would snap back or say I’m not interested or something else”. (client 1)

Her ability to name and differentiate multiple emotions, such as sadness, happiness, satisfaction, and fear, suggests an increased capacity for emotional awareness. By identifying these emotions in real-time, she established a clearer connection between her internal state and external validation, demonstrating a shift toward emotional self-recognition and trust in her own abilities.

Client 4, on the other hand, described how she assigned meaning to her experiences through both emotions and bodily sensations, using them as a lens for interpreting past situations:

“And so ah very few headaches, where before I had been extremely sore. So that’s almost gone [after therapy]. Every now and then I think to myself, okay, headache again, the weather, I don’t know, but before that it was because I was so tense due to the situation and had a lot of headaches as a result, exactly. And of course, it feels much better now. Yes ah easier”. (client 4)

Her awareness of bodily sensations as indicators of emotional distress suggests a shift in how she processes physical symptoms. She acknowledged that her headaches were previously linked to tension and emotional strain, but now she experiences a sense of ease and relief, demonstrating a newfound ability to interpret bodily signals in relation to emotional well-being.

These findings highlight how clients engage with their emotions and bodily sensations as part of their therapeutic progress. They have developed a more integrated understanding of their internal experiences through greater emotional awareness and meaning making. Observing these changes over time allowed them to recognize the impact of therapy on their emotional and physical states, reinforcing their ability to navigate emotional challenges with increased self-awareness and confidence. These changes were observed in clients with low integrated personality structure displayed in two PETs with eight subthemes for Client 1, one PET with five subthemes for Client 4, and one PET with seven subthemes for Client 2 with moderate integration.

#### 3.4.2 Relating and reflecting on the past and present

This subtheme highlights the process of connecting past experiences to current behaviors, emotions, and relational patterns.

Client 2, for example, used self-reflection as a tool to detach from unhealthy family dynamics, gaining a greater understanding of how her familial relationships influenced her sense of self:

“It’s also challenging for me to admit to myself that I can also be without [family], that I have this family support. There are already structures in my family that are totally stressful for me and where I have the feeling that I’ve somehow been keeping myself down for a very long time or perhaps being kept down”. (client 2)

Here, her struggle to separate from family structures suggests a deep-rooted internal conflict between the security of familial bonds and the recognition of their restrictive nature. Her words “keeping myself down” and “being kept down” indicate both self-imposed and external limitations, reflecting a growing awareness of how these relational patterns have shaped her self-perception and autonomy.

She further reflected on how her past shaped her emotional responses and patterns of coping, realizing that seeking comfort in familiar environments did not necessarily fulfill her emotional needs:

“And then I coped for a very long time, that I went to my parents’ house quite regularly at the weekend, and then I felt relatively - well. That was very pleasant for a while because I was back in this familiar childlike behavior, but then I soon realized that this feeling of loneliness wasn’t satisfied there either”. (client 2)

By recognizing that returning to childhood patterns did not resolve her loneliness, she demonstrated an evolving ability to critically assess past and present emotional experiences, marking a step toward greater independence.

Similarly, client 3 reflected on how childhood experiences of caretaking and conflict mediation continued to shape her relational dynamics in adulthood:

“But as a child I always tried to mediate somehow between my parents and to appease somehow and calm them down, and that was exhausting as a child, as an adult it is of course, a bit easier, but when you try to help your parents somehow for decades and it just doesn’t work because they just can’t get along, oh well, that’s a bit, but that was as a child”. (client 3)

Her statement reveals a longstanding role as a mediator in family conflicts, which extended into her adult life. The phrase “for decades” underscores the enduring emotional burden of this role, illustrating how deeply embedded relational patterns from childhood can persist into adulthood. While she acknowledged that handling these situations “as an adult is of course a bit easier,” her continued struggle with unresolved family dynamics suggests an ongoing process of emotional differentiation and detachment.

Both clients had moderately integrated personality structures and used self-reflection to explore the emotional and relational impact of their childhood experiences. However, through reflection, the moderate-integrated client gained deeper insight into how early life events, relationships, and unresolved issues shaped her present experiences. This self-awareness enabled her to recognize recurring patterns, develop emotional distance from dysfunctional relationships, and become more attuned to her personal needs. By identifying these repetitive patterns, she was able to gain emotional distance from past struggles, reassess her current relationships, and gradually redefine her sense of self beyond the constraints of their early experiences. Overall, these patterns were observed only in clients with higher integration, as shown in three PETs with 15 subthemes for Client 2 and five PETs with 29 subthemes for Client 3.

## 4 Discussion

This study explored how four clients with suboptimal structural integration experienced their navigation through challenging situations and decision-making since they have been in Gestalt therapy. By employing Interpretative Phenomenological Analysis (IPA), we examined their subjective experiences, highlighting some potential differences between individuals with low- and moderate-integrated personality structures. Given the small sample size, these findings are not intended to be generalized but rather an exploratory perspective on how clients with varying levels of personality integration have navigated the therapeutic process and how specific internal mechanisms could support or hinder more profound structural change. In doing so, we consider the role of narrative identity, relational engagement, and structural self-organization in shaping therapeutic experiences.

### 4.1 Narrative identity and meaning-making in therapy

Gestalt therapy’s emphasis on present-moment awareness appeared to support the clients in this study in different ways. The two clients with low integration engaged with therapy primarily through bodily awareness, using physical sensations and emotional immediacy to anchor their experiences (see also 30). While they became more adept at identifying and verbalizing emotions, they struggled to integrate these insights into a broader, cohesive self-narrative. Their emotional awareness remained largely experiential rather than interpretative, meaning that they recognized emotions in the moment but did not connect them to past experiences or future perspectives. This aligns with the idea that bodily awareness may serve as a foundational step for clients with weaker personality integration before more complex cognitive and relational integration can occur (28). However, bodily awareness alone did not foster sustained structural change, as these clients continued to rely on external validation for emotional regulation rather than developing internal coherence.

The two clients with moderate personality integration engaged more actively in reflective meaning-making, linking past experiences to present challenges and attempting to integrate their emotions into a coherent self-narrative. However, while they demonstrated an increased ability to recognize relational and emotional patterns, these insights often remained intellectual rather than fully internalized. That is, they could articulate meaning but struggled to embody it in a way that led to stable self-assurance. This suggests that while meaning making is essential in therapy, its effectiveness in fostering agency depends on the extent to which clients integrate their insights into their lived experience. This distinction was particularly evident in how clients navigated relational challenges: those with lower personality integration continued to rely on external validation, whereas those with moderate personality integration showed greater attempts at autonomy, albeit with lingering self-doubt.

Constructing a coherent self-narrative is considered a key component of structural integration (19). The findings suggest that clients with low integration experienced difficulties in creating continuity between past and present experiences, often perceiving emotions as fragmented or disconnected from prior relational patterns. Rather than linking past difficulties to a broader sense of self, their focus remained on immediate emotional states, reinforcing a fragmented self-experience. This aligns with Fuchs (19) argument that disturbances in narrative identity can lead to a disconnected sense of self, making it challenging to establish a stable self-concept:

“Splitting and fragmentation of the self creates an ever-new present, an isolated now that is separated from the past and the future” (19, p. 210).

### 4.2 Relational dynamics in Gestalt therapy

Relational experiences were central to the therapeutic processes described by the clients in this study, but their engagement with relationships differed based on their level of structural integration. Clients with low integration relied heavily on others for emotional stabilization and decision-making, frequently seeking external validation and describing feelings of helplessness when relational support was unavailable. This finding aligns with research suggesting that individuals with lower personality integration often depend on external figures to regulate their emotional states (38, 39). While OPD-2 (2014) approaches these dynamics through structured diagnostic frameworks, especially in analyzing and discussing relationship patterns and psychodynamic conflicts, relational psychoanalysis focuses on fluid, co-created intersubjective experiences (40, 41). Both OPD-2 manual and relational psychoanalysis emphasize the therapeutic significance of exploring relational disruptions in therapy. In Gestalt therapy, relational aspects are grounded in a dialogic and mutual field that highlights contact, awareness, and emotional co-regulation between client and therapist (25, 35). The therapist takes on the role of an active co-experimenter and authentic presence, engaging in a genuine, mutual dialogue (26, 42), thereby creating opportunities for new and corrective relational and bodily-felt experiences for the client. However, given the small sample size, these observations remain tentative.

The two clients with moderate integration also sought relational validation but appeared to use it more flexibly. Rather than relying on others for emotional regulation, they engaged in relationships as a way to affirm their perceptions and reinforce decision-making. However, their continued self-doubt, particularly in situations requiring assertiveness, suggests that relational autonomy remained a challenge.

These findings relate to Buber’s concept of self-development through interpersonal connections (2017), emphasizing that meaningful relationships shape self-perception and agency. Furthermore, the self is not an isolated entity but is continuously shaped by bodily, social, and ecological contexts (43). Individuals actively construct meaning through embodied interactions with their environment, which plays an integral role in shaping personal identity and understanding of the world. Intersubjectivity, therefore, is not merely a cognitive process but is deeply rooted in embodied social interactions (28). This perspective aligns with clients’ descriptions of therapy as a process in which relational and bodily awareness played a key role in their evolving self-understanding.

### 4.3 Gestalt therapy and self-development

Our findings further link to Gestalt therapy concepts of self-development (44, 45), particularly in terms of forming a coherent, independent, and stable self. From a Gestalt perspective, the mature self develops through early relational experiences, forming *Daseinsgewissheit*, a foundational certainty of existence, which enables *Selbstgewissheit* a sense of self-assurance (17, 46). Deficits in relational experiences may contribute to an unstable self, marked by identity instability and difficulties in emotional regulation (15, 47, 48). These characteristics were evident in the self-perceptions of the two clients with low integration in this study.

The two clients with low integration described increased emotional awareness but continued to struggle with self-cohesion and relational autonomy. Their reliance on external co-regulation remained unchanged, suggesting that while therapy facilitated emotional awareness, structural self-organization remained limited. Clients making corrective experiences and improvements on personality functioning might require long-term psychotherapy.

The two moderate-integrated clients showed some capacity for independent coping but remained reliant on relationships for emotional support. They connected past experiences to present challenges, yet their self-narrative lacked full cohesion (15, 48). From a Gestalt perspective, their self-assurance was supported by primary ego functions, which shaped their perceptions of others (17, 49). While they faced challenges in achieving full cohesion and autonomy, they found support in positive relationships. Therapy helped them engage in reflective processing, yet self-doubt persisted in situations requiring independence.

### 4.4 Limitations and future directions

As an Interpretative Phenomenological Analysis (IPA) study, this research prioritized depth over breadth, focusing on how a small, relatively homogenous group of female clients with suboptimal structural integration made sense of their experiences after 30 sessions of Gestalt therapy. IPA does not aim for statistical generalizability but instead seeks to generate rich, idiographic insights into specific lived phenomena (32, 50). In this case, the phenomenon concerns how clients navigate emotional and relational challenges, bodily awareness, and agency in the context of therapy. While this design allowed for an in-depth exploration of these experiences, the findings are not intended to represent all clients with common mental disorders or varying levels of personality integration. Rather, they offer theoretical insights that may inform our understanding of similar therapeutic processes in comparable settings.

Future studies could expand on these findings by incorporating a larger sample. This would allow for a broader range of perspectives while maintaining the idiographic depth of IPA. While gender homogeneity was a methodological choice, future research could explore whether similar patterns emerge in male clients.

Another consideration is the timing of data collection. Retrospective accounts allowed participants to articulate their experiences with some reflection, but future studies could conduct multiple interviews at different points in therapy to examine how clients navigate through processes of change as they unfold. While this would not constitute a traditional longitudinal study, it could provide a more nuanced view of structural integration over time.

### 4.5 Conclusion

While acknowledging its limitations, this study suggests that clients with suboptimal structural integration may rely on bodily awareness and external validation to navigate self-perception, emotional regulation, and meaning making in Gestalt therapy. Clients with low integration primarily engaged with therapy through bodily awareness and external validation but struggled to develop a coherent self-narrative. In contrast, clients with moderate integration demonstrated reflective capacity and relational reinforcement but continued to experience self-doubt and difficulties with autonomy. These findings indicate that while emotional awareness and external support may facilitate growth, more profound structural transformation could require additional therapeutic factors, particularly for clients with lower integration.

Common therapeutic factors across psychotherapy approaches include the establishment of a strong therapeutic alliance, the provision of a safe and supportive relational environment, client hope and expectation, and opportunities for emotional expression and insight (51–53). These elements form a foundation for change regardless of the specific modality. Gestalt therapy, while sharing many of these common elements, emphasizes unique therapeutic processes such as awareness in the present moment, authentic contact between therapist and client, and the co-creation of the therapeutic relationship through dialogic engagement (14; 26, 35). This focus on the here-and-now experience and the mutual field enables clients to increase self-regulation and experiment with new ways of being in relational contexts, facilitating corrective emotional experiences that extend beyond cognitive insight (25). Thus, Gestalt therapy’s specific factors highlight the dynamic, experiential, and relational nature of therapeutic change.

Future research should further investigate how clients with varying levels of structural integration develop self-efficacy and internalized self-assurance. Additionally, clinical interventions may need to extend beyond emotional regulation and relational autonomy to target the consolidation of self-cohesion. For clients with lower integration, this may involve gradually shifting reliance from external validation toward more stable internalized processes of self-organization and meaning making.

This study highlights important clinical considerations for working with clients who exhibit varying levels of structural personality integration in Gestalt therapy. Clients with low integration primarily benefit from increased bodily awareness and emotional immediacy with the therapist, which serve as foundational steps toward emotional regulation through experimentation in the here-and-now. However, their efforts to form a coherent self-narrative often depend heavily on external validation to maintain emotional stability. In contrast, clients with moderate integration demonstrate greater capacity for reflective meaning-making and relational engagement, linking past experiences to present challenges. Yet, their insights often remain intellectual rather than fully embodied, and self-doubt – particularly around autonomy and assertiveness – may persist. For these clients, therapy may focus on deepening the integration of cognitive and emotional insights into lived experience, thereby strengthening self-efficacy and relational autonomy.

## Data Availability

The original contributions presented in the study are included in the article/**Supplementary Material**. Further inquiries can be directed to the corresponding author.
