# Supplementary material for: Clients’ subjective experiences of navigating challenges in Gestalt therapy: an interpretative phenomenological analysis
Source: Front Psychiatry. 2025 Oct 27;16:1593918. doi: 10.3389/fpsyt.2025.1593918 (PMC12597916; doi:10.3389/fpsyt.2025.1593918)
Supplement: Supplementary file 1 [file Table1.docx]

Supplementary Material

| **Supplement Table 1. Overview of Personal Experiential Themes (PET) of all clients.** | | | | |
| --- | --- | --- | --- | --- |
| CLIENT 1 | CLIENT 2 | CLIENT 3 | CLIENT 4 |  |
| **Theme 1: Ideal conception of oneself and others** | **Theme 1: Connecting the Present Situation to the Past** | **Theme 1: External Impulses as Triggers for Decisions** | **Theme 1: Low Self-Worth and Negative Perception of Men** |  |
| Ideal conception of other families and children, others make no mistakes | Compares current friendships with past experiences to make decisions | External impulse as a trigger for decisions | Self-deprecation (others are more competent) |  |
| Ideal idea of what a mother has to do to offer her children something | Compares current emotions to past feelings | Seeks external permission not to do something | Fear of judgment and criticism of her opinions |  |
| Ideal idea of how she should behave in conflicts | Links feelings of loneliness to past challenging relationships | Hopes that the partner will decide differently | Low confidence in her own opinions |  |
| Doubts about the ideal | Endures and finds meaning in loneliness | Input from others provides more security and relief | Low self-esteem, doesn't take herself seriously |  |
| **Theme 2: Negative self-image and self-worth** | **Theme2: Self-Assurance and Security Through Positive Resonance from Others** | The body as its own instance making decisions (the "good body") | Negative perception of men |  |
| Feeling of hopelessness/helplessness due to emotional shoas (‘permanent puberty’) | Transition from co-regulation to self-regulation through change | Recognizes being overwhelmed and refrains from acting | Communicates boundaries externally but disregards her own |  |
| No confidence in themselves and their own opinion | Self-care is now possible (post-romantic relationship) | Seeks reassurance in therapy on how to act (external impulse) | Escape and avoidance as a way out of threatening situations |  |
| Positive experience and feedback from team is weakened by generalisation | Perspective shifts provide security | Deals with guilt by outsourcing decisions to bodily impulses | **Theme 2: Reliance on Others' Opinions for Decision-Making (Co-Regulation)** |  |
| Generalisation (as a woman) serves to compare with the ideal and distance oneself from one's own performance | Reduced pressure in voice messages/responses creates safety | **Theme 2: Overlooking Own Needs** | Struggles to make conscious decisions independently |  |
| **Theme 3: Negative anticipation of situations** | Resonance from others provides relief and self-confidence | Doesn't express her feelings in conversations (they go unnoticed) | Relying on others' opinions provides security and minimizes errors |  |
| Devaluation in comparison with other families in fictitious situations | Self-assurance and trust in the world through resonance offer security | Ignores the cost of taking on a mediator role | Daughter’s approval supports decision-making |  |
| Anticipates negative experiences in advance | **Theme 3: Self-Doubt and Insecurity in Social Interactions** | Differentiating feelings is more challenging ("feels weird") | Justifies her opinions to others |  |
| Fear of appointment and reactions of others, as something new | Social interactions are intense and challenging | Feels like she’s missing out when not participating in everything | Justifies decisions by taking minimal risks (avoiding bad outcomes) |  |
| Negative anticipation of support from the family with regard to decision-making | Insecurity in social interactions, not learned | **Theme 3: Emotional Distance from Parents** | **Theme 3: Devaluation of Others as a Justification for Her Own Decisions** |  |
| Negative anticipation if children would not agree | Fear of being hurt, leading to avoidance of friends | Awareness of progress in emotion regulation | Devalues others to justify her decisions |  |
| **Theme 4: Devaluation of successful situations** | Self-doubt causes insecurity | Better able to classify her emotions through therapy | Criticizes men to help accept her decisions |  |
| Devaluation of the successful situation | Concealing as an expression of insecurity about identity | Emotional distance aids in self-regulation | Experiences the importance of decisions (major decisions) as a sign of something new or transformative |  |
| Devaluation of the positive experience as an evaluation of the situation | Needs validation of personal needs from others | Fluent, clear, and coherent storytelling through emotional detachment | Criticism of her opinions prompts reevaluation and builds self-assurance |  |
| Explanation model for success is chance (no personal contribution) | **Theme 4: Voice Messages as a Strategy to Cope with Insecurity** | Feels calm and physically relaxed | **Theme 4: Downplaying Threatening Situations Through Generalization Leads to Inaction** |  |
| Even if the family succeeds at something, the other families can do it better (nerd parents) | Distance in voice messages through time delay, location difference, and impersonal nature | **Theme 4: Relating Experiences to Childhood** | Downplays the severity of threatening situations |  |
| **Theme 5: External impulses as (co-)regulation** | Voice messages provide immediate relief | As a child, hoped her parents would resolve their conflicts through her mediation | Relativizes threatening situations by comparing herself to other women |  |
| External stimulus changes the mood, cannot be controlled by itself (co-regulation) | Voice messages as a safe way to connect with friends (new strategy) | As an adult, realizes her parents' relationship doesn’t work | Downplays positive outcomes by generalizing them |  |
| Plans of others are placed in the foreground | Voice messages as a form of self-determination in actions | Reflects on her role as a mediator in childhood, which was exhausting | Minimizes her own needs compared to others |  |
| Participation in the organisational development process by others | **Theme 5: Detailed Perception of Bodily Sensations and Emotions After Change** | Connects current experiences in conversations to past experiences with her parents | Succumbs to external pressure and adapts |  |
| **Theme 6: Relieving collective decisions** | Agonizing, inhibiting state when alone (before change) | **Theme 5: Parentification in Childhood and Taking Responsibility for Parents** | **Theme 5: Reinterpreting Threatening Situations Causes Helplessness and Inaction** |  |
| Shared responsibility brings relief, a sense of happiness and flexibility in actions | Describes emotions through sensations, finds naming emotions difficult | Took care of her parents as a child (one-on-one talks) | Reinterpreting threatening situations leads to helplessness |  |
| Shared responsibility in a collective relieves the burden and brings relief | Detailed description of feelings (loneliness) | Co-regulated her parents' intense emotions | Uses reinterpretation to justify inaction |  |
| Group has confidence in them and their decisions | Distinct bodily awareness during depressive phases (low, unmotivated, powerless; pressure on the chest, lump in the throat) with associated meaning | Didn’t learn to focus on herself (too much responsibility) | The threat of involving the police provides subjective protection |  |
| Re-evaluation is positive and brings calm through collective decisions | Acceptance of feelings (loneliness) diminishes loneliness | Identifies with her mother (also sensitive) | Explains tolerance for the situation as having a high threshold |  |
| Support from superiors enables participation in the organisational development process | Experiences physical relief (lump becomes smaller) | Protects her father from being overwhelmed, as in childhood | **Theme 6: Knowledge and Reflection Provide Self-Assurance** |  |
| **Theme 7: Pressure to make your own decisions** | Opens up to others, feels less shame post-change | Downplays childhood experiences (not idyllic) | Expertise and knowledge help contextualize threatening situations |  |
| Making the decision yourself creates pressure | **Theme 6: Recognizing Own Needs Facilitates Change** | Struggles to say no to avoid hurting others | De-idealizes friends during decision-making |  |
| Decision-making by holiday destination as recurring pressure | Behavioral changes by focusing on own needs | Uses sensitivity as a framework to explain her emotional responses | Awareness of her own boundaries helps contextualize the situation but doesn’t lead to consequences |  |
| Deciding early and spontaneity relieves pressure | Change enables inner freedom and curiosity to explore | Overwhelmed by responsibilities, such as organizing a wedding (too much) | Reflecting on past relationships aids in understanding the situation |  |
| No support from the family regarding the decision | Felt fatigue with old patterns, leading to change | Takes responsibility for her father, shielding him | Through reflection, the threat is reassessed |  |
| **Theme 8: Self-confidence and knowledge enable own decision-making** | Positive experiences in social interactions inspire confidence | Has been taking responsibility and experiencing parentification since childhood | Finds a way out of the situation through self-reflection and focusing on herself |  |
| Feedback from the family helps in the decision-making process | Has hope of finding her identity | **Theme 6: Relief Through Parental Resonance** | Develops self-assurance and confidence over time, leading to different decisions (explanatory framework) |  |
| Own decisions can be made on the basis of knowledge | After the change, sharing burdens with friends becomes a helpful and effortless process | Finds relief as her father takes care of himself after the separation | **Theme 7: Assigning Meaning to the Situation Through Emotion and the Body** |  |
| Satisfaction from others' trust in them when making decisions | **Theme 7: Uncertainty in Social Connections** | Feels relief through her mother acknowledging her emotions | Provides a detailed description of bodily sensations during positive situations |  |
| Once a decision has been made, pressure is released | No certainty that challenging situations can be faced together | Proud and relieved that she no longer feels responsible for her parents’ relationship | Perceives situations as less threatening when physical and emotional sensations become evident, bringing relaxation |  |
| **Theme 9: Differentiation of emotions and bodily emotions in successful situations** | Previously avoided loneliness by visiting parents | Supports her mother with more emotional distance than before | Understanding meaning through emotion and the body brings a sense of ease |  |
| Admitting that sometimes something does succeed | Emergency strategy: going to parents | **Theme 7: Mediator Role in Conversations with Parents** | Relaxation on both emotional and physical levels gives new meaning to the situation |  |
| Emotional differentiation when reflecting on the situation | Seeks co-regulation in social connections (initially family) | Uses childhood examples to help her parents empathize with situations | Stabilizes through emotional detachment in positive situations, creating lightness |  |
| Excitement before a situation can be emotionally differentiated with a description of bodily sensations | Looks for social contact during lonely moments (being supported) for validation | Applies insights from therapy to illustrate the dynamics between her parents |  |  |
| Leaving the situation in case of conflict as an important strategy for impulse control | Feels ashamed of her poor state, leading her to turn to family | Aware that certain physical positioning during conversations signals taking sides |  |  |
|  | **Theme 8: Dependence on External Circumstances Driving Change** | Takes on a new role as an adult mediator rather than a substitute partner |  |  |
|  | Doesn't view her new strategies as her own success (they just happened) | Acts as a mediator in conversations instead of a caregiver for one parent |  |  |
|  | Doesn't equate self-care with support from others | Balances a mediator role between her parents |  |  |
|  | Doesn't see change as her own achievement (met new people and attended therapy) | Adopts a moderator role in conversations to highlight dynamics and create distance (meta-level) |  |  |
|  | Feels a dilemma between family and loneliness; change wasn't consciously initiated | **Theme 8: Self-Reflection and Giving Meaning to Actions** |  |  |
|  | Past relationship distracted her from loneliness by understanding needs and family dynamics | Questions automatic behaviors (e.g., sitting next to her mother) |  |  |
|  | Relationship hindered self-care as the partner needed much support | Prioritizes her own needs in situations |  |  |
|  | External factors (summer, COVID, others’ sociability) contributed to improvement, not personal efforts | Generalizes emotions, feeling general relief in life due to her parents’ separation |  |  |
|  | Dilemma: desires change but feels simultaneously driven and restrained | Evaluates conversations as positive |  |  |
|  | **Theme 9: Generalization Helps Justify Behavior** | Reflects on the meaning of gestures in situations (e.g., remaining neutral) |  |  |
|  | Normalizes her behavior through generalization (applies to everyone) | Reflects on her behavior and takes action (e.g., changes seating position) |  |  |
|  | Holds normative beliefs, such as friendships involving no arguments | Regulates her emotions in the moment |  |  |
|  | Grieving process automatically eases difficult situations over time | **Theme 9: Self-Doubt and Insecurity** |  |  |
|  | Generalizes that people are reluctant to call, normalizing her own behavior | Anticipates negative reactions, leading to inaction |  |  |
|  | **Theme 10: Self-Reflection Creates Emotional Distance from Dysfunctional Relationships and Detachment** | Takes a long time to share even small examples |  |  |
|  | Reflects on a long process of coping with loneliness | Downplays her significant role in conversations (calls it "a bit of moderating") |  |  |
|  | Gained courage to face personal issues through reflection on a past relationship | Feels uncertain and seeks reassurance about whether she answered questions correctly |  |  |
|  | Emotional distance and detachment through reflection on family dynamics and needs | Doubts whether her parents understand the situation |  |  |
|  | Experiences less emotional emptiness and physical discomfort after change | Questions her ability to regulate emotions |  |  |
|  | Ending the grieving process (relationship) allows acknowledgment of personal needs | **Theme 10: Support in Decision-Making Eases Pressure** |  |  |
|  | Strategy existed before the difficult phase and was rediscovered | Shocked that her partner decided not to have children |  |  |
|  | **Theme 11: Detachment Process (Becoming an Adult) as a Dilemma** | Can tolerate ambivalence (an open decision) |  |  |
|  | Growing up is a long, arduous path she must walk alone | Not making a decision eases pressure |  |  |
|  | Explanatory model: the complex world and finding grounding during adulthood | Makes conscious, active decisions after external prompts |  |  |
|  | Complexity of the world as a framework for slow identity formation | Avoiding decisions reduces pressure in situations |  |  |
|  | Concealing as a dilemma: authenticity vs. being alone (burdening friends with personal weaknesses) | Receives support for decision-making in therapy |  |  |
|  | Weighs possible consequences for decision-making | Weighs possible consequences for decision-making |  |  |
|  | Treats major life-changing decisions (e.g., children) with great care | Treats major life-changing decisions (e.g., children) with great care |  |  |

| **Supplement Table 2. Group Personal Experiences Themes of Gestalt therapy process** | | |
| --- | --- | --- |
| Themes | Line | Quotes |
| **Group Experiential Theme 1: Self-concept of idealisations and devaluation of others** |  |  |
| Client 1: Ideal conception of oneself and others | 13 | So there are always crises within my family ... well, I mean my children are in puberty now and I can't accept that. |
| Client 4: Devaluation of others as a justification for her own decisions | 29 | That's how quickly you get into it [stalking]. Suddenly you don't know what to do anymore, exactly. |
| Client 4: Reinterpreting threatening situations causes helplessness and inaction | 27 | So I counsel women who are affected by violence and I've seen that I always have to say to them ‘I'm not so badly affected myself’ but in a different form. And now you're in there so quickly. |
| Client 2: Generalization helps justify behavior | 140 | But it doesn't make the other person have to react immediately [to voice messages] [...] and somehow people are now a bit inhibited about making phone calls, I have the feeling. |
| **Group Experiential Theme 2: Self-perception** |  |  |
| **Theme 2a: Devaluing oneself and others (black and white thinking)** |  |  |
| Client 1: Negative self-image and self-worth | 17 | And I have the feeling that I myself am in a state of permanent puberty. And I often don't know what I want today will be different tomorrow or I'll talk myself into something or talk myself out of something, so it's actually getting more and more bizarre what I think. And then I really have to go away and let them do it because otherwise it escalates [family situation]. |
| Client 1: Negative anticipation of situations | 60 | So it was like, let's say, 30 people were contacted [project] to see if they wanted to take part and then I don't know, four or five people got in touch to continue working on the topic [...] and ahm, no one winded me up or anything else because I just got in front of them and I actually did very well. And what's also nice is that I started by thinking I was afraid of the appointment because maybe all the unpleasant people would get in touch or maybe ahm I don't know uh write back then go back please I'm not interested in that. But I was able to get a few people motivated and I think maybe they wouldn't have done that with everyone. |
| Client 1: Devaluation of successful situations | 50, 52 | [...] but I mean, these are the kind of things where I thought ah yes, I mean it was a coincidence [borrowing toiletries to niece] that it was in the bag or the bag that I just had with me. But then I thought to myself actually well done and it was definitely needed in the situation. As a woman, you have to be prepared. |
| Client 4: Low self-worth and negative perception of men | 146 | I know my way around and I'm interested, but some things I just don't check in politics. So in a way, yes ah, I've realised that I just don't feel comfortable, so to speak, with people who say ‘you can't do that [vote]’ [...] that I used to think that he and she are much more knowledgeable and read better articles, so that's why I have to attach myself to them, so to speak [opinion] |
| Client 4: Devaluation of others as a justification for her own decisions | 167 | You talk about it and realise that others only boil with water. |
| **Theme 2b: Self-doubt and insecurities** |  |  |
| Client 2: Self-doubt and insecurity in social interactions | 58 | I ahm still don't want to impose this [problems] and that I don't want to impose myself on others, and that I make myself vulnerable with my weakness) or with my issues, which are somehow present there, and I wasn't quite sure how that works [getting in touch]. |
| Client 2: Uncertainty in social connections | 192 | I really thought, well, I have to pretend so that people stay with me so that I don't feel lonely and on the other hand I can't show myself because otherwise they run away and that's why I'm lonely and that I have been lonely for a very long time or that I still find it difficult to be honest with myself and to maintain honest relationships. |
| Client 2: Detachment process (becoming an adult) as a dilemma | 309 | Things need time [to grow up]. That's really difficult for me ahm because I somehow often and very often have the feeling that I'm always behind [being independent] and that I'm so slow, but things can take time. |
| Client 3: Self-doubt and insecurity | 120 | I think that would actually be too much for the bride too [party], but somehow I don't dare say that it's too much, because it might come across as weird or something, ahm, and then somehow I didn't really know what to do about it |
| **Group Experiential Theme 3: Relieving yourself through others** |  |  |
| **Theme 3a: Navigating decision-making by external input** |  |  |
| Client 1: External impulses as (co-)regulation | 23 | There was a chat message from a group and we met up at a place, that was a 20-year reunion, and the chat message was full of positive vibes, as they say, and then I thought to myself, don't let it be ruined somehow and just go [away from the conflict situation]. |
| Client 1: Relieving collective decisions | 99 | When you visit a city [with your family], you don't always have to decide for yourself what to do. So that was also a big weight off my shoulders, that everyone should like it now |
| Client 1: Self-confidence and knowledge enable own decision-making | 129, 135 | I asked the children if they had any big ahm, if they had any big ah ah what's it called ahm, if they had any big objections to this holiday destination, and after nothing came and nobody said ah [...] I suggested the destination, and two years ago I actually said in March or April, when we were still working from home, that we would do it spontaneously, again because I said we would book something now and stay in Austria. |
| Client 4: Reliance on others' opinions for decision-making (Co-Regulation) | 146 | I allowed myself to be distracted or made dependent on the opinions of people who I really appreciate and who know their stuff very well. |
| Client 4: Knowledge and reflection provide self-assurance | 43 | How do you get into [a stalking situation] yourself? So maybe it was mainly about reflecting on that. So about the guy himself, yes, it wasn't really about him any more, yes ah, but about how do I get into a situation like that as a woman. |
| **Theme 3b: Resonance from others is helpful to make own decisions** |  |  |
| Client 2: Voice messages as a strategy to cope with insecurity | 146 | I think I'm even more uninhibited because I don't have this direct feedback [from the other person]. I can just chat freely, and if I get a voice message in return, I can choose when I listen to it and when I reply to it, and then I can organise my own resources a bit better. |
| Client 2: Self-assurance and security through positive resonance from others | 129 | [...] there are people and they see me and I can communicate with them, and that's okay, and I can do that and I can be who I am. |
| Client 3: External impulses as triggers for decisions | 174 | I had wanted to start therapy for a long time and then I just couldn't bring myself to do it. And then there was another dicey situation with my parents, and then I thought, okay, actually I like my life the way it is anyway, and I don't want to end my life just because it's always difficult, and then I went shopping and looked to the right, and then I saw that there was a therapy practice, and then I thought, okay, well, that's fate now. |
| Client 3: Overlooking own needs | 27 | She [mum] almost started crying for a moment [conversation about separation of parents], but then pulled herself together, and I was almost a bit shaken, and then I said no, and didn't cry. Anyway, the conversation was kind of real, I tried to moderate it a bit, and then I also kind of looked to the left and right and then I often talked about what I'd learnt in therapy. |
| Client 3: Relief through parental resonance | 27 | At the end of the conversation, my mum took me in her arms, thanked me for the conversation and then apologised because she had finally realised that my childhood wasn't so rosy, she always knew, but for various reasons she couldn't do it any other way or couldn't look after me the way she might have wanted to, and she apologised. |
| Client 3: Support in decision-making eases pressure | 158 | My boyfriend doesn't want to have children at the moment and we've actually been together for a little over four years now and we've always said that we want to have children and then somehow, out of the blue, he said to me at some point that he couldn't actually imagine having a child because of the climate crisis and all the other crises in the world. That was a slap in the face for me and then we somehow decided to discuss it in therapy and there's no decision yet on what to do next. |
| **Group Experiential Theme 4: Therapy progress** |  |  |
| **Theme 4a: Experiencing in the here and now** |  |  |
| Client 1: Pressure to make your own decisions | 127 | So that was a lot of pressure [holiday planning], because at first I wanted to go to a country next to Ukraine, and then I wanted to visit my sister, who lives outside Europe, and then it was a bit like maybe I won't be there after all, and I have to go there, but now that she's coming to Austria, I thought well then I'll see it anyway or we'll see her anyway and then we spontaneously decided on a third country because I saw a documentary on TV. |
| Client 1: Differentiation of emotions and bodily emotions in successful situations | 78 | Ahm emotion, ahm ahm so excitement is an emotion? No. There's a certain sadness that I can't go in more confidently, that's there a priori. Then there's a certain feeling of happiness that I managed to send the email, there's a bit of satisfaction that when I said I'd take care of it, no one said, ah yes you hm so it was the same yes yes do it. [...] that's a kind of satisfaction that there's already a trust in me now. [...] and of course I was afraid that someone would snap back or say I'm not interested or something else. |
| Client 4: Assigning meaning to the situation through emotion and the body | 71 | And so ah ah very few headaches, where before I had been extremely sore. So that's almost gone, for example. Every now and then I think to myself, okay, headache again, the weather, I don't know, the weather, but before that it was because I was so tense due to the situation and had a lot of headaches as a result, exactly. And of course it feels much better now. Yes ahm ah easier. |
| Client 2: Detailed perception of bodily sensations and emotions after change | 112 | I often felt so down, somehow lacking in energy and drive. Yes, and I also often had pressure on my chest and a lump in my throat, so somehow everything closed up and everything was hard. Somehow at the same time a feeling of not being able to and at the same time a feeling of being driven by something that has to be different and somehow it's wrong and I feel that it shouldn't be like this but I can't actually get out of it. |
| **Theme 4b: Relating and reflecting on the past and present** |  |  |
| Client 2: Self-Reflection creates emotional distance from dysfunctional relationships and detachment | 239 | It's also challenging for me to admit to myself that I can also be without ahm, that I have this family support. Because I don't know, there are already structures in my family that are totally stressful for me and where I have the feeling that I've somehow been keeping myself down for a very long time or perhaps being kept down. |
| Client 2: Recognizing own needs facilitates change | 245 | I really have the feeling, and I can totally feel it, that a lot has simply changed in the last year, especially in the last six months. And that I'm dealing a lot with myself and with my own issues and my own fears and worries and that I'm trying to understand them better somehow. |
| Client 2: Connecting the present situation to the past | 16 | And then I copied for a very long time or ahm, that I went to my parents' house quite regularly at the weekend, and then I was relatively, well, that was very pleasant for a while because I was back in this familiar childlike behaviour a bit, but then I soon realised that this feeling of loneliness wasn't satisfied there either. |
| Client 3: Self-reflection and giving meaning to actions | 57 | So now it's a difficult phase again [between the parents], and then of course I support them, but somehow I try not to get as involved as perhaps I used to [...] just by putting myself in the middle [in the conversation with parents], luckily that came to me, because otherwise it would probably have been stupid, well that would have just felt strange [...] although at that moment it was just totally automatic, but I just thought to myself, well that doesn't fit, and I just got up [and sat between the parents]. |
| Client 3: Relating experiences to childhood | 89 | But as a child I always tried to mediate somehow between my parents and to somehow appease and calm them down, and that was exhausting as a child, as an adult it is of course a bit easier, but when you try to help your parents somehow for decades and it just doesn't work because they just can't get along, oh well, that's a bit, but that was as a child. |
| Client 3: Parentification in childhood and taking responsibility for parents | 84 | Because then I somehow explained [to the parents] that dad comes home, is overwhelmed, and then mum comes and wants to load things up with him and that's just too much for him, ahm, and I just used that as an example [them] to somehow explain how they might feel. I don't know whether it's because they felt that way or because they understood it. |
| Client 3: Mediator role in conversations with parents | 91 | I just tried to see the conversation between my parents from a bit of a bird's eye view, or just from a moderator's point of view, and not to get involved as a child, but simply to guide them through the conversation a bit. |
| Client 3: Emotional distance from parents | 39 | And such a charged atmosphere [in the conversation with the parents] and so on, somehow already partly charged by my parents, but I actually remained quite matter-of-fact, and then somehow always tried to calm them down a bit when I realised that they were getting louder or more emotional, ahm, and tried to counteract that somehow |
